# Muscular coordination of biceps brachii and brachioradialis in elbow flexion with respect to hand position

**DOI:** 10.3389/fphys.2015.00215

**Published:** 2015-08-06

**Authors:** Tim Kleiber, Leo Kunz, Catherine Disselhorst-Klug

**Affiliations:** ^1^Department of Rehabilitation and Prevention Engineering, Institute of Applied Medical Engineering, RWTH Aachen UniversityAachen, Germany; ^2^German Research School for Simulation Sciences, Joint Graduate School of RWTH Aachen University and Forschungszentrum JülichJülich, Germany

**Keywords:** pronation, supination, sEMG, muscular activation, elbow flexor, inter-muscular coordination

## Abstract

Contribution of synergistic muscles toward specific movements over multi joint systems may change with varying position of distal or proximal joints. Purpose of this study is to reveal the relationship of muscular coordination of brachioradialis and biceps brachii during elbow flexion with respect to hand position and biomechanical advantages and disadvantages of biceps brachii. A group of 16 healthy subjects has been advised to perform 20 repetitions of single elbow flexion movements in different hand positions (pronated, neutral, and supinated). With a speed of 20°/s, simultaneously sEMG of biceps brachii and brachioradialis and kinematics of the movement were recorded in a motion analysis laboratory. Normalized to MVC the sEMG amplitudes of both muscles contributing to elbow flexion movements were compared in pronated, supinated, and neutral hand position over elbow joint angle. Significant differences in the contribution of brachioradialis were found in pronated hand position compared to supinated and neutral hand position while the muscular activity of biceps brachii shows no significant changes in any hand position. In conclusion, a statistical significant dependency of the inter-muscular coordination between biceps brachii and brachioradialis during elbow flexion with respect to hand position has been observed depending on a biomechanical disadvantage of biceps brachii.

## Introduction

Inter-muscular coordination of synergistic and antagonistic muscles can be regarded as the basis to explain the generation of voluntary and target-oriented movement. Biomechanics and muscular features contributing to human movement patterns are thereby combined to control inter-muscular coordination and optimally recruit the responsible muscles. Moreover, insights into neural commands and a better understanding of motor control and muscular coordination can contribute to improve diagnosis and treatment of both, neuromuscular dysfunction and resulting orthopedic conditions, or vice versa.

The elbow joint is a highly complex joint assembled by three different single joints (Amis and Miller, 1982). As a connection between upper arm and forearm a special focus has to be set on the forearm complex which consists of two bony parts, radius and ulna. This joint complex offers the possibility to move in two degrees of freedom, flexion and extension as well as pronation and supination. During elbow flexion the forearm is moved toward the upper arm rotating around the elbow joint center. Pronation and supination are performed by radius and ulna crossing each other and so rotating forearm and hand to a maximum of 90° from neutral hand position. There are different muscles involved in elbow flexion which are superficially biceps brachii and brachioradialis as well as deeper brachialis. Both superficial flexors are also involved in other functions and movements of connecting joints e.g., biceps brachii is also supinator and shoulder flexor, brachioradialis is responsible for both, supination as well as pronation to move the forearm back in neutral position (Deetjen and Speckmann, 1999).

The function of brachioradialis and its contribution to elbow flexion as well as pronation and supination has been and is still discussed with diverging results (Jackson, 1925; Sullivan et al., 1950; de Sousa et al., 1961; Pauly et al., 1967; An et al., 1981; Funk et al., 1987; van Bolhuis and Gielen, 1997; Naito, 2004; Boland et al., 2008). One hypothesis of Jackson is, that brachioradialis changes its contribution to elbow flexion with hand position which has been proved through experiments (Jackson, 1925; Praagman et al., 2010). Boland et al. published no differences in contribution of brachioradialis during elbow flexion in different hand position and varying external forces and so concluded that it is mainly stabilizing the elbow joint, which directly stands in contrast to earlier studies (Stokes and Gardner-Morse, 2000; Boland et al., 2008). Nakazawa et al. investigated the contribution of brachioradialis during concentric and eccentric elbow flexion resulting in significant differences of the muscular activation pattern to finally consider brachioradialis primary as an elbow flexor especially in lower joint angles supporting the point of view of Jackson and Boland (Howard et al., 1986; Nakazawa et al., 1993). Additionally, a speed dependent activation of brachioradialis with higher contribution in elbow flexion in higher velocity is stated (de Sousa et al., 1961).

Purpose of this study is to reveal the relationship of inter-muscular coordination of biceps brachii and brachioradialis during elbow flexion movements with respect to hand position. A special focus is set on biomechanical advantages and disadvantages of biceps brachii influencing an optimized recruitment strategy of both muscles. Based on the results of Boland et al. there are no changes in the contribution of brachioradialis in elbow flexion depending on hand position. But there may be a reasonable explanation of an occurring difference due to the biomechanical disadvantage of biceps brachii in pronated hand position.

## Material and methods

### Subjects

A sample of data of 16 healthy subjects [4 female and 12 male, age 24.8 (±9.2) years; height 179.4 (±9.9) cm; body mass 79.1 (±8.8) kg] took part in the study. No subject had any known symptoms of neuromuscular disorders or orthopedic surgery or affections in the upper extremity. Subjects avoided strenuous exercises in the day prior to the measurement. 14 out of 16 subjects were right handed and all subjects were in a comparable training state. The study was performed according to the Declaration of Helsinki and approved by the RWTH Aachen University ethic committee. All subjects were informed about the experimental protocol and the potential risks of the study and gave written consent before their participation.

### Study protocol

The measurements took place as non-clinical basic science study in a motion analysis laboratory. Single trials of dynamic elbow flexion movements were performed fluently in a constant speed of 20°/s guided by a visual feedback of the joint angle. The elbow flexion movement was repeated 20 times for each hand position separately in single trials from full extension (approx. 0°) to maximal flexion (approx. 130°). There was a resting time of 120 s after each trial to avoid fatigue. Subjects were measured in standing position with shoulder neither flexed nor abducted. The different hand positions were measured in neutral and each subject's maximum of pronation and supination.

### Kinematics and sEMG data acquisition

A specific marker setup presented in Schmidt et al. (1999) was used to record kinematics during movement via VICON® MX motion analysis system. Elbow joint angles were determined by a biomechanical model using the marker setup described above (Williams et al., 2006). Infrared light reflecting markers (9 mm diameter) were placed on six anatomical landmarks of the upper extremity (acromion, olecranon, radial styloid process, ulnar styloid process, epicondyle lateral, epicondyle medial). Markers were attached using double-sided adhesive tape. Joint centers of elbow and wrist are estimated as the midpoint between both epicondyles and styloid processes, respectively. Three rigid linked markers, called triplets, were placed on the upper body segments (thorax, upper arm, forearm, hand). Through the exact position of recorded segment marker triplets relative to computed elbow joint center, determined on static calibration trials, all joint angle positions are synchronously measured to the sEMG recordings of biceps brachii and brachioradialis (Rau et al., 2000). Through the recording of the whole kinematic chain compensatory movements in the shoulder joint an wrist which may also influence the sEMG amplitude (e.g., shoulder flexion, changes in hand position) can be excluded (Schmidt et al., 1999; Williams et al., 2006).

**Figure 1:**
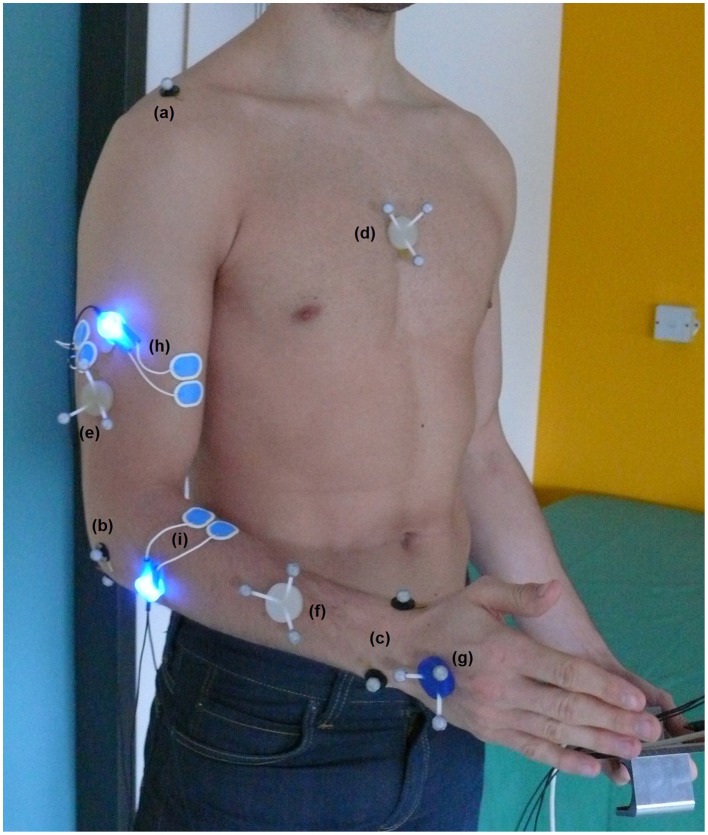
**Full marker setup for kinematic analysis of upper extremity including joint marker of acromion (a), elbow joint (b) and wrist (c) as well as marker triplets on the segments thorax (d), upper arm (e), forearm (f), and hand (g)**. Bipolar sEMG electrode placement including pre-amplifier with blue LEDs of biceps brachii (h) and brachioradialis (i).

Bipolar sEMG signals of biceps brachii and brachioradialis muscle are recorded and processed according to standard protocols developed with the SENIAM recommendations (Hermens et al., 2000). Single-use Ambu® Blue Sensor N electrodes (effective electrode diameter of 3 mm) were placed in a distance of 2 cm on the muscle belly directly connected to a pre-amplifier (blue LED). The full marker setup and electrode placement is shown in Figure 1.

Processing steps of the recorded sEMG signals after 3 kHz sampling include pre-filtering (lower cut-off frequency 2 Hz, upper 500 Hz), full-wave rectification and smoothing (Root Mean Square, window length 100 ms).

### Normalization via MVC

For a proper comparison of activation levels of both muscles intra-individually special focus must be set on the standardization of the sEMG signals (Burden, 2010). To put the activation levels of both muscles in relation to each other, reference values of both flexors were recorded during 5 s maximum voluntary isometric contraction measurements. Since sEMG amplitudes vary for different elbow angles, sEMG signals are standardized with the maximal amplitude in 90 degrees elbow flexion. Out of five MVC trials the mean of the best three with a minimum standard deviation and no significant differences in the maximal amplitude were chosen. MVC was determined separately for every hand position and all signals were normalized to the according maximal value to account the contribution of muscle to hand position.

### Statistical analyses

Statistical significance was determined by One-Way analysis of variance (ANOVA) with a significance level of *p* < 0.05. Main effects for each independent variable were investigated, and test statements were used to specify error terms. A Tukey HSD *Post-hoc* and Students *t*-Test were additionally used to specify the results. Statistical results were interpreted relative to biomechanical and biological significance.

## Results

**Figure 2:**
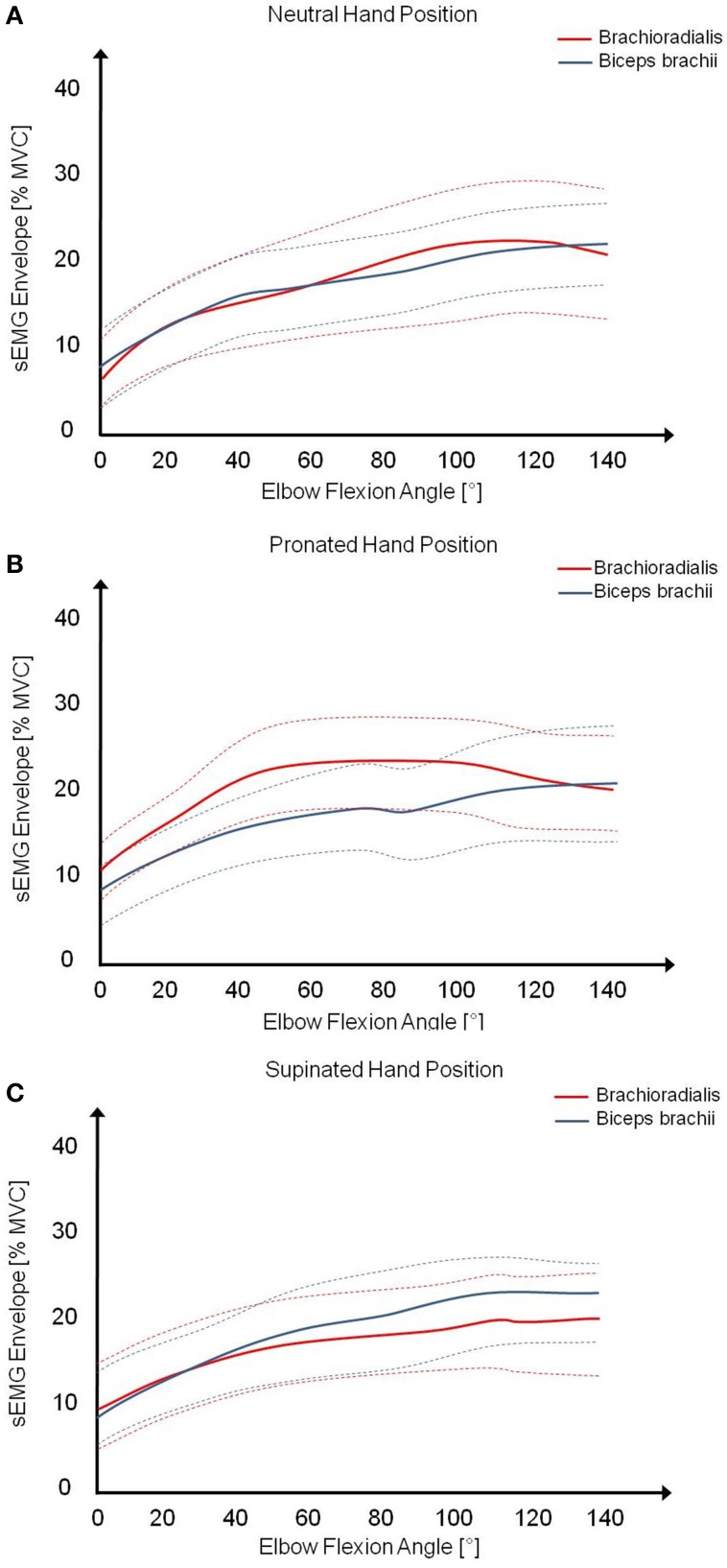
**Mean (solid lines) and standard deviation (dashed lines) of the muscular activity of biceps brachii and brachioradialis from all subjects during elbow flexion in (A) neutral, (B) pronated, and (C) supinated hand position**.

The acquired and processed sEMG data of biceps brachii and brachioradialis were assigned over measured elbow joint angles and analyzed in the range between 0 and 120° during concentric elbow flexion. The mean (solid lines) and standard deviation (dashed lines) of all subjects were calculated for both muscles in different hand positions, shown in Figure 2. Examining the muscular activity normalized to MVC, there are obvious differences in muscular coordination pattern during pronated elbow flexion in comparison to supinated and neutral hand position. As in pronated, the muscular activity of brachioradialis is constantly higher than in supinated and neutral hand position whereas the percentage activity of biceps brachii is nearly the same in all hand positions. The muscular activity of both muscles is constantly on the same level and slightly increasing with elbow joint angle in supinated and neutral hand position.

**Figure 3:**
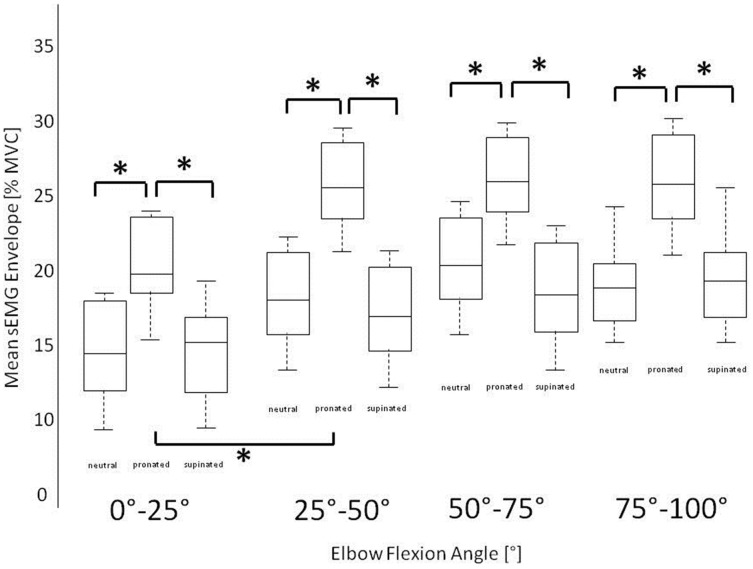
**Stepwise mean normalized sEMG amplitude of brachioradialis in three different hand positions in intervals of 25° of elbow flexion joint angle showing statistical significant differences in pronated hand position in comparison to neutral and supinated hand position**.

Statistical analysis shows significant differences (^*^) in muscular activation pattern of brachioradialis during elbow flexion with respect to hand position (*p* < 0.05). The *Post-hoc* Tukey Test shows not only a general difference between the activation patterns of both elbow flexors with respect to hand position but additionally a significantly higher difference in pronated than in supinated and neutral hand position while the activity of biceps brachii remains constant. Between supinated and neutral hand position there were no significantly differences in muscular coordination pattern of both muscles (biceps *p* = 0.75, brachioradialis *p* = 0.67). In biceps brachii there are no significant differences (*p* = 0.63) in any hand position. Figure 3 shows the mean value of the activation level of brachioradialis in steps of 25° of elbow joint angle in all three different hand positions. Significant differences are found in pronated in comparison to supinated and neutral hand position in all considered intervals.

## Discussion

Purpose of this study was the investigation of differing muscular contribution of biceps brachii and brachioradialis during elbow flexion with respect to hand position finding a reasonable explanation in a biomechanical disadvantaged role of biceps brachii as an elbow flexor in pronated hand position. The function of brachioradialis has been discussed in literature with diverging results (de Sousa et al., 1961; Nakazawa et al., 1993; Boland et al., 2008). The presented results agree with most of the authors in the fact that brachioradialis is an active flexor of the elbow with increasing contribution in pronated hand position (Jackson, 1925; de Sousa et al., 1961; Howard et al., 1986; Nakazawa et al., 1993; Praagman et al., 2010). Hereby it is important to consider that there may be an influence in brachioradialis' recruitment strategy depending on the biomechanical disadvantaged role of biceps brachii in pronation. Therefore only an observation of the muscular activity of both muscles may lead to a useful interpretation.

The results show clearly the function of brachioradialis as elbow flexor with a significant increased contribution in pronated hand position. This can be concluded from the presented sEMG measurement in pronated hand position compared to neutral and supinated hand position, whereas the activation level of biceps brachii remains constant in all three hand position. From a biomechanical view brachioradialis has a longer anatomical lever arm than biceps brachii. Consequently less muscular force than in biceps brachii is required to hold an external weight. However because of the longer lever arm brachioradialis demands a stronger contraction to flex the elbow and so a biomechanical disadvantage takes place. So brachioradialis function is mainly lifting or holding an external weight, including the weight of the forearm like stated in Frisch (2000) and de Sousa et al. (1961). But in pronated hand position the biceps tendon is wrapped by its insertion in tuberosida radii (Howard et al., 1986; Deetjen and Speckmann, 1999). Considering this fact, there is a biomechanical disadvantage of biceps brachii in pronated hand position to flex the elbow and the biomechanically advantaged brachioradialis takes over a higher contribution in elbow flexion because less muscle force can be generated by biceps brachii due to the disadvantaged lever arm at a constant activity. These circumstances result in a significantly higher activity of brachioradialis to compensate the lower torque produced by biceps brachii although the activation level of biceps brachii is the same like in supinated and neutral hand position.

It should also be considered that there is neural inhabitation of biceps brachii and brachioradialis as stated in Naito et al. (1996). This may explain the similar activation level of both muscles in supinated and neutral hand position.

The often discussed contribution of brachioradialis to pronation and supination cannot be proved by this study. It should be mentioned that because of the normalization of the sEMG amplitudes to the specific MVC in every hand position, the contribution of biceps brachii and brachioradialis to pronation and supination movements are canceled in the processed normalized signals. So neither biceps as a supinator nor brachioradialis as a pronator/supinator can be examined here.

## Conclusion

There is a strong influence of hand position on the inter-muscular coordination of biceps brachii and brachioradialis in elbow flexion. This has been shown by a significant increased muscular activity of brachioradialis during elbow flexion in pronated compared to supinated and neutral hand position whereas activity of biceps brachii remains constant. This change in contribution of brachioradialis can be reasonable explained by the biomechanical disadvantaged role of biceps brachii in pronation resulting in brachioradialis taking a higher contribution in elbow flexion.

### Conflict of interest statement

The authors declare that the research was conducted in the absence of any commercial or financial relationships that could be construed as a potential conflict of interest.
